# Design and Validation of Probes and Sensors for the Characterization of Magneto-Ionic Radio Wave Propagation on Near Vertical Incidence Skywave Paths

**DOI:** 10.3390/s19112616

**Published:** 2019-06-09

**Authors:** Ben A. Witvliet, Rosa M. Alsina-Pagès, Erik van Maanen, Geert Jan Laanstra

**Affiliations:** 1 Centre for Space, Atmospheric and Oceanic Science (CSAOS), Department of Electronic and Electrical Engineering, University of Bath, Claverton Down, Bath BA2 7AY, UK; 2 Telecommunication Engineering (TE), Faculty of Electrical Engineering, Mathematics and Computer Science, University of Twente, P.O. Box 217, 7500AE Enschede, The Netherlands; 3 Grup de recerca en Tecnologies Mèdia (GTM), La Salle-Universitat Ramon Llull, Quatre Camins, 30, 08022 Barcelona, Spain; rosamaria.alsina@salle.url.edu; 4 Spectrum Management Department, Radiocommunications Agency Netherlands, P.O. Box 450, 9700AL Groningen, The Netherlands; erik.vmaanen@agentschaptelecom.nl; 5 Data Science–Data Management & Biometrics (DS/DMB), Faculty of Electrical Engineering, Mathematics and Computer Science, University of Twente, P.O. Box 217, 7500AE Enschede, The Netherlands; g.j.laanstra@utwente.nl

**Keywords:** deployable, magneto-ionic, magnetic field, polarization, fading, ionosphere, radio wave propagation, Near Vertical Incidence Skywave (NVIS), circular polarization

## Abstract

This article describes the design and validation of deployable low-power probes and sensors to investigate the influence of the ionosphere and the Earth’s magnetic field on radio wave propagation below the plasma frequency of the ionosphere, known as Near Vertical Incidence Skywave (NVIS) propagation. The propagation of waves that are bent downward by the ionosphere is dominated by a bi-refractive mechanism called ‘magneto-ionic propagation’. The polarization of both downward waves depends on the spatial angle between the Earth’s magnetic field and the direction of propagation of the radio wave. The probes and sensors described in this article are needed to simultaneously investigate signal fading and polarization dynamics on six radio wave propagation paths. The 1-Watt probes realize a 57 dB signal-to-noise ratio. The probe polarization is controlled using direct digital synthesis and the cross-polarization is 25–35 dB. The intermodulation-free dynamic range of the sensor exceeds 100 dB. Measurement speed is 3000 samples/second. This publication covers design, practical realization and deployment issues. Research performed with these devices will be shared in subsequent publications.

## 1. Introduction

**Figure 1:**
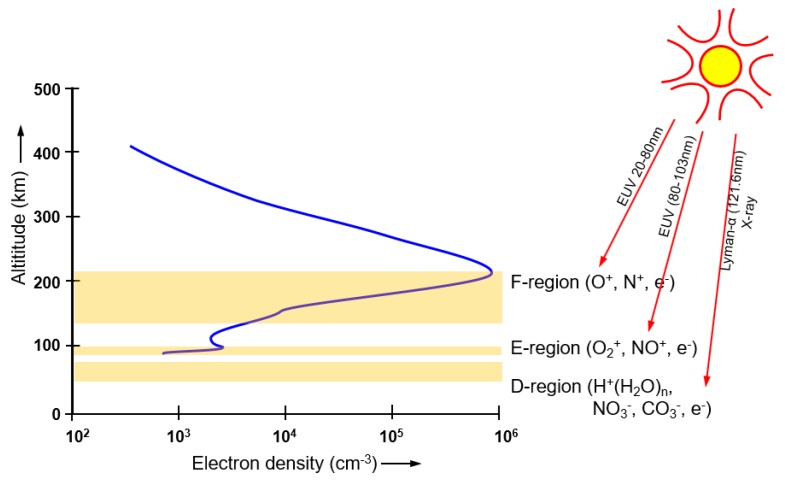
The ionosphere is a natural plasma, sustained by solar radiation [4].

**Figure 2:**
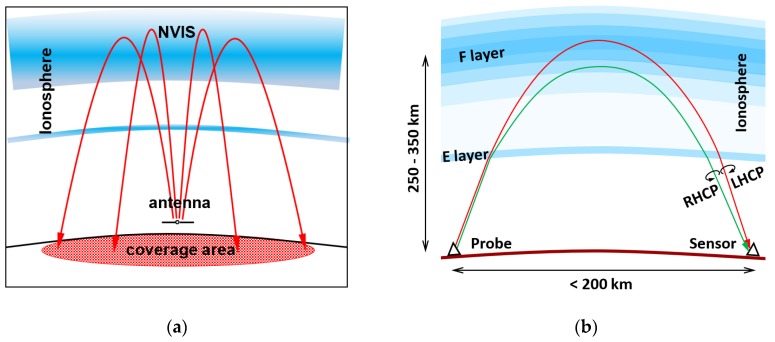
(**a**) Near Vertical Incidence Skywave (NVIS) propagation; (**b**) Magneto-ionic propagation: ordinary (red) and extraordinary wave (green). Polarization is shown for the northern hemisphere.

When a natural disaster occurs, relief work is severely hampered by telecommunication infrastructure damage. This is best illustrated with the landfall of Hurricane Katrina and the subsequent flooding of New Orleans in 2005 [1]. Most of the telecommunication infrastructure was destroyed by the storm [2]. Given that the electricity distribution was also disrupted, the few remaining cell towers operated on backup batteries for another 4 h, after which they ceased to work as well [3]. The roads into the disaster were flooded, which left the entire disaster zone, an area of 200 km × 200 km, without access and telecommunications. In such circumstances, as an alternative, ionospheric radiocommunication systems could be used to provide instantaneous access to the entire area. The ionosphere is a natural plasma between 60 and 1000 km height, sustained by solar radiation [4]. Several regions can be distinguished [4], as shown in Figure 1. The peak electron density occurs between 150 and 250 km height in the F-region. Ionospheric radio systems may send radio waves nearly vertically upwards, to be refracted in the ionosphere and returned to earth, as depicted in Figure 2a. This phenomenon is called ‘Near Vertical Incidence Skywave’ (NVIS) propagation [5]. The refraction in the ionosphere depends on the electron density in the ionosphere [6]. Driven by the radiation of the sun, the electron density follows a diurnal cycle, the seasons and the 11-year solar cycle [7]. For NVIS propagation, the frequency of the radio waves must be smaller than the maximum plasma frequency of the ionosphere, for mid-latitudes typically between 3 and 10 MHz.

The ionosphere is bi-refractive. Appleton and Builder showed that radio waves entering the ionosphere, under the influence of the Earth’s magnetic field, are split in two circularly polarized characteristic waves in opposite rotational directions, the ordinary and the extraordinary wave [8]. Appleton and his contemporaries [9,10] were able to develop this ‘magneto-ionic theory’ based on work of Maxwell and Thomson [11], that explained the polarization rotation discovered by Faraday in 1845 [12]. The polarization of the characteristic waves depends on the angle between their direction of propagation and the magnetic field vector. In the northern hemisphere, the polarization of the ordinary wave is right-hand circular (RHCP) upwards and left-hand circular (LHCP) downwards, as shown in Figure 2b. In the Southern hemisphere, the sense of rotation is reversed. For NVIS propagation at mid-latitudes and at frequencies above 5 MHz, the polarization of the characteristic waves is almost circular [6]. Both characteristic waves follow a different path through the ionosphere, which can be shown using ray-tracing techniques [13,14]. And as the ionosphere is not homogeneous, they suffer different attenuation, time delay and delay spread, Doppler shift and Doppler spread and fading.

### 1.1. Previous Work

NVIS radio systems were used in New Orleans, but not to their full potential. Most practical organizations refer to the book of Fiedler and Farmer [5] on NVIS, which only provides generic information. A large volume of NVIS research exists, but it is scattered over 70 years and a wide range of media. To improve access to it, the authors published an overview article on NVIS that refers to 128 NVIS publications, organized per subject [15]. To support work from other groups that optimize High Frequency (HF) antennas for mobile platforms [16,17,18] and fixed installations [19,20]. The authors provided elevation angle measurements and raytracing simulations [21] to extend graphs published by McNamara [22] and Davies [6]. These now include the influence of frequency and sunspot number. Furthermore, the authors showed that the bi-refractive properties of the ionosphere could be used to transmit two isolated waves on the same frequency for diversity and Multiple-Input Multiple-Output (MIMO) [23]. Erhel et al. demonstrated their application in an HF MIMO system [24]. Precise measurements of the authors showed that the isolation of the characteristic waves in the ionosphere is at least 25–35 dB [25] and they described the ‘Happy Hour’ propagation phenomenon for the first time. Nearly perfectly circular polarization was observed on a 105-km-long NVIS path from north to south at 52.7°N. Night-time observations on frequencies above the maximum usable frequency (MUF) were reported earlier by Wheeler and McNamara [26,27]. The authors showed that, at night-time above the critical frequency, the received waves become unpolarized and exhibit fluttery fading [28]. Very similar characteristics were observed when a solar X-ray flare inhibited the NVIS path between transmitter and receiver [28]. 

### 1.2. Research Goals

Our previous research also identified new areas where important questions remained. Firstly, all our measurements were performed in the Netherlands at 52.7°N, with a magnetic dip angle of 69°, on a north-to-south path. Nearly perfectly circular polarization was observed in 2 different NVIS experiments spaced seven months apart [25,28]. To test the generality of these observations and of the isolation between the characteristic waves, measurements over multiple azimuths are needed, preferably repeated at a second location with a different magnetic dip angle. Furthermore, pronounced fading was observed in NVIS experiments in which the transmitted and received polarization were matched to one of the characteristic waves, which was unexpected [28]. 

Our current research therefore has three main objectives: (i) Verification of the isolation between both characteristic waves for different azimuths, distances and magnetic dip angles; (ii) Simultaneous measurement of the polarization dynamics on multiple radio wave paths with different azimuths and distances; (iii) Analysis of signal fading and its possible origins, by means of comparing the radio wave propagation on all paths. 

The first goal is the focus of the project. It will validate the previous measurements conducted in the Netherlands [25] and confirm the hypothesis that the achievable data rate can be doubled using characteristic wave propagation. The second goal is relevant for (possibly dynamic) optimization of antenna polarization, which will improve the isolation between the ordinary and the extraordinary waves. The third goal is perhaps the most ambitious: although signal fading is common in HF propagation, the proposed study will expand the knowledge on its mechanisms and drivers, and enable future mitigation techniques.

### 1.3. The Need for Novel Probes and Sensors

For each of these objectives, specialized equipment is needed to empirical obtain data of the radio wave propagation and the resulting radio channel. Duplicating our previous measurement system is not practical because of its size and the costs involved. System designs could not be adopted from other groups either: Hervás et al. [29] and Walden [30] did not measure polarization. Erhel et al. used large fixed antennas and did not achieve sufficient cross-polarization [24]. We therefore decided to design a novel system of probes and sensors that is portable (<20 kg), low-power (<5 W), quickly installed (<2 h) and autonomous. The sampling speed will be increased from 0.5/s to 3000/s. For this design we were able to build on the experience gained in our previous experiments. This publication covers the system design, practical realization and deployment criteria. 

The article is structured as follows: The experiment with one central sensor and multiple probes is described in Section 2. Frequency selection for the experiment is described in Section 3. The design of the central sensor is detailed in Section 4. The sensor specifications and link budget calculations are provided in Section 5 are then used to specify and design the probes in Section 6. Section 7 deals with deployment and Section 8 validates the design in a field deployment. The article concludes with a discussion and suggestions for future work.

## 2. Description of the Experiment

A network of probes and sensors will be created for these investigations. The probes will emit electromagnetic waves of constant frequency towards the ionosphere. When these waves are refracted back to earth, the sensor will measure their polarization and signal strength and store these data for postprocessing. From them, the mean path loss and fading dynamics can be calculated, as well as mean polarization, polarization dynamics, Doppler shift and Doppler dispersion. To investigate polarization effects, the probing signals will be switched periodically between LHCP, RHCP and linear polarization. 

**Figure 3:**
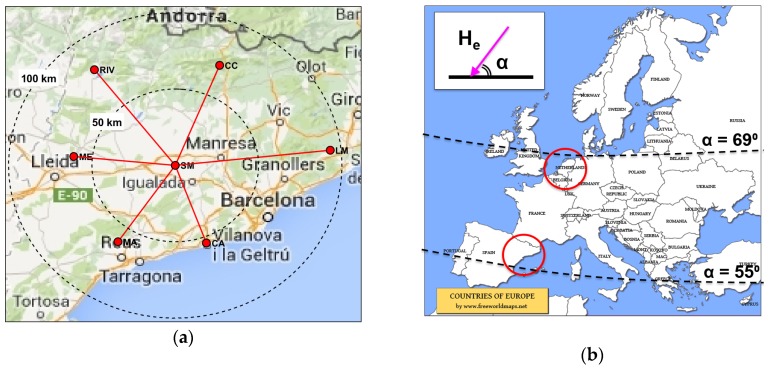
Experiment topology: (**a**) An example of a star-shaped deployment (Catalonia, Spain); (**b**) The inclination of the Earth’s magnetic field changes with latitude. Map courtesy of Free World Maps.

Our previous experiments were all performed on a north-south path in The Netherlands (52.7°N, 6.4°E) over a ground distance of 105 km. For more generalized conclusions, the experiment must be extended to cover multiple azimuths and distances, to obtain different angles of the radio waves with the magnetic field. Therefore, in this experiment, measurements will be performed on waves emitted by 4–8 probing signal transmitters deployed in a star-like arrangement around a single very accurate sensor, as shown in Figure 3a. To avoid interference between groundwave and skywave, the probes are located at a distance of between 50 and 200 km from the sensor. At distances beyond 20–30 km distance, at frequencies above 5 MHz, the ground waves will be sufficiently attenuated and the sky waves will dominate [21].

The experiment will be repeated at two different locations in the Netherlands (52.21°N, 5.04°E) and Spain (41.68°N, 1.48°E). This is important as the inclination of the Earth’s magnetic field changes with latitude, as shown in Figure 3b. The International Geomagnetic Reference Field (IGRF) model describes the direction and magnitude of the Earth’s magnetic field [31]. In the Netherlands, at a height of 220 km in the ionosphere, the inclination and declination are respectively 66.9° and 0.9°. In Spain, they are 56.7° and 0.2°. When varying the azimuth angle between 0° and 360° and the distance between 50 and 150 km, the angle between the downward waves and the Earth’s magnetic field will vary from 2° to 48° in the Netherlands and from 8° to 58° in Spain.

The repetition of the experiment in two countries requires transportable probes and sensors. The installation of six probes in an area with a radius of 100 km requires 900 km of driving. Therefore, the installation of the entire system on a single winter day is only feasible when the work is divided between two teams and the installation time is less than 2 h per probe. To eliminate the need for staff supervision of the equipment, automation is essential. And as the best measurement locations are away from buildings and structures, battery operation and low power consumption are required. Furthermore, as multiple probe signal transmitters are needed, cost of production and calibration become important. As high accuracy is required as well, novel solutions are necessary. 

## 3. Frequency Selection

The frequency at which the experiments will take place has to be considered first, because of its significant impact on the design. On one hand, the sensor can only measure the signal of the probes when NVIS propagation is present for a large part of each day. This puts an upper limit on the frequency used. Lowering the frequency, on the other hand, will increase the absorption in the D-region and the ambient electromagnetic noise (radio noise) level and therefore require significantly more probe power. A lower frequency also implies a larger antenna, which will make the deployment more difficult. For NVIS propagation the peak plasma frequency of the ionosphere must be higher than the frequency of the electromagnetic wave. The local plasma frequency is directly related to the local electron density in the ionosphere [32] and the highest frequency at which the ordinary wave is reflected vertically is:

(1) $$f_{oF2}=\frac{1}{2\pi }\sqrt{\frac{e^{2}N_{max}}{\varepsilon_{0}m_{e}}}\approx 8.98\sqrt{N_{max}}$$

where *f_oF2_* is the critical frequency in Hz, *N_max_* the peak electron density in electrons/m^3^, *e* the charge of an electron (1.602 × 10^−19^ C), *ε_0_* the permittivity of free space (8.854 × 10^−12^ F/m), and *m_e_* the mass of an electron (9.109 × 10^−31^ kg). The critical frequency *f_xF2_* of the extraordinary wave is slightly higher [32]:

(2) $$f_{xF2}=f_{oF2}\sqrt{1+{\left(\frac{f_{H}}{2f_{oF2}}\right)}^{2}}+\frac{f_{H}}{2}$$

where *f_H_* is the electron gyrofrequency in Hz, which ranges from 1.0 to 1.2 MHz at 220 km height in the Netherlands and Spain, so that Equation (2) can be approximated as:

(3) $$f_{xF2}\approx f_{oF2}+0.55\;\mathrm{MHz}$$

**Figure 4:**
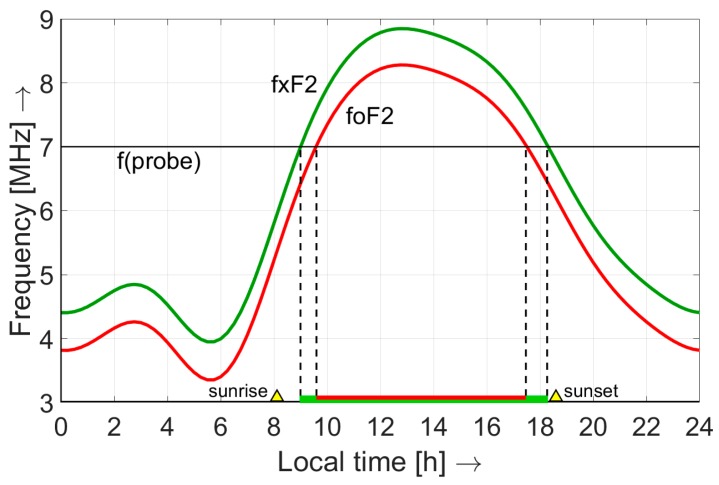
Modelled diurnal variation of the critical frequencies of the F2-region of the ionosphere using the International Reference Ionosphere [34]. NVIS propagation is possible when *f_xF2_* > *f_probe_*.

There is no propagation when *f_xF2_* is lower than the probe frequency *f_probe_*. Only the extraordinary wave propagates when *f_xF2_* > *f_probe_* > *f_oF2_*. And both waves propagate when *f_oF2_* > *f_probe_*. The electron production in the ionosphere is driven by solar radiation [4] and therefore *f_oF2_* and *f_xF2_* follow a diurnal cycle, increasing in the morning when the sun starts to illuminate the ionosphere and decreasing in the afternoon, as shown in Figure 4. In this example, NVIS propagation is possible from 09:00–18:00 h. The minimum and maximum values depend on the location on Earth, the day of the year and the position in the 11-year solar sunspot cycle [7]. The experiments were planned in the winter to take advantage of the higher electron densities caused by the ‘winter anomaly’ in the northern hemisphere [33]. After analysing ionospheric sounding data from the Royal Observatory of Belgium (50.1°N, 4.6°E) and the Ebre Observatory in Spain (40.8°N, 0.5°E), the critical frequency *f_oF2_* was expected to remain above 7 MHz during at least 8 h of each day. Therefore, an experimental license was obtained in the Netherlands to use a clear frequency of around 6.99 MHz. In Spain, the experiments were conducted around 7.04 MHz using an amateur radio license. To enable simultaneous observation of all probes, each of them was given a small (110 Hz) frequency offset. Unmodulated probes signals were used in the measurements. With sufficient frequency stability the sensor will separate them by frequency filtering. Narrow-band filtering will also increase the signal-to-noise ratio (SNR) and allow for lower probe power. We therefore choose a sensor bandwidth of 30 Hz.

## 4. Sensor Design

A sensitive sensor will allow all the probes to emit weaker signals, and thereby significantly simplify their design, reduce size and cost and improve transportability. Typically, at frequencies below 20 MHz, the ambient electromagnetic background noise (or radio noise) limits the maximum sensitivity that can be achieved [35] (p.766). Electrical appliances generate strong impulsive electromagnetic noise, but even in a quiet rural environment radio noise will be observed, caused by the accumulative emissions of distant thunderstorms and cities arriving via ionospheric reflection. Therefore, for our experiment it is important to deploy the sensor in a quiet rural environment, where the radio noise level is low. Furthermore, the noise floor of the sensor has to be lower than the expected radio noise level, which can be found in ‘Recommendation ITU-R P.372’ [36] (Equation (13)). For a quiet rural location, it predicts the radio noise level as:

(4) $$F_{amb}=53.6-28.6\;{log}_{10}f$$

where *F_amb_* is the ambient noise factor in dB and *f* is the frequency in MHz. Using this Equation, we find that *F_amb_* is 29 dB at 7 MHz, from which the noise power at the antenna terminals *P_n_* can be calculated [36] (Equation (6)):

(5) $$P_{n}=F_{amb}+10\;{log}_{10}\left(kT_{0}b\right)$$

where *P_n_* is the noise power in dBW, *k* is Boltzmann’s constant (1.38 × 10^–23^ J/K), *T_0_* is the reference temperature, of 290 K and *b* is the bandwidth in Hz. Assuming a lossless antenna, *P_n_* is expected to be −160 dBW in 30 Hz bandwidth. To achieve maximum sensitivity, the noise floor of the sensor should be at least 10 dB lower than the external noise level. At the same time the sensor must be very linear, to avoid intermodulation products of strong shortwave signals. Transmissions from high-power shortwave broadcast stations at distances between 500 and 2000 km may produce a cumulative input power of −70 dBW. Therefore, the intermodulation free dynamic range (IFDR) must exceed 100 dB. This requirement is challenging even for most professional HF receivers. 

The sensor consists of a transducer (antenna) and a sampler (receiver). The antenna converts the electromagnetic wave into electrical signals, and the receiver converts the HF signal to baseband and stores 3000 samples/second on hard disk. A hardware platform designed by the open source ‘High Performance Software Defined Radio’ (HPSDR) group is used [37]. It has 2 coherent 16-bit analogue-to-digital convertors (ADC) sampling at 122 MS/s. Measured amplitude and phase drift are less than 0.01 dB and 0.2° over 48 h during field deployment. The signals are filtered in the digital domain, converted to baseband and stored as 32-bit complex samples. Polarization is calculated from the data of both input ports and RHCP, LHCP and linear polarizations can be derived simultaneously on a per-sample basis. 

**Figure 5:**
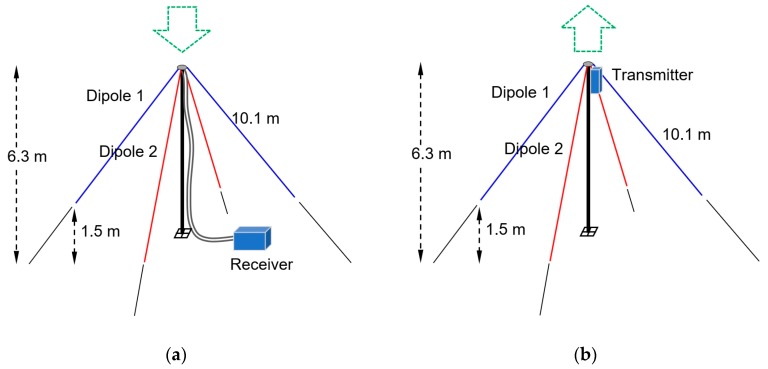
(**a**) The sensor; (**b**) One of the probes. Distance between probe and sensor is 50–200 km.

A full-size passive antenna has been chosen as a transducer, to retain linearity and achieve a high efficiency. It consists of two resonant Inverted Vee dipoles made of stranded copper wire. Each of them has linear polarization. By mounting two of them perpendicular and connecting them to the two coherent receiver inputs, full polarization can be measured on a per-sample basis. This design yields a cross-polarization of 25–35 dB. The dipole leg length L can be calculated as:

(6) $$L=\frac{c}{4f}.v$$

where *c* is the speed of light (2.9979 × 10^8^ m/s), *f* is the frequency in Hz and *v* is the relative wave velocity along the antenna wire, which is approximately 0.95 for a wire diameter of 1 mm. For a frequency of 7.04 MHz, the leg length is approximately 10.1 m. The antenna, shown in Figure 5a, has a footprint of 25 × 25 m. The antenna was modelled using NEC4.2 Method of Moments software with a realistic Sommerfeld ground model [38]. The antenna gain of a single element towards the zenith is 3.7 dBi. Equal length coaxial feedlines connect the two antenna elements to the dual-channel receiver. The same antenna will be used for the probe, but now connected to a dual channel transmitter unit mounted in the masthead, as shown in Figure 5b.

## 5. Link Budget Calculations

From previous section we know that sensitivity of the sensor, limited by radio noise, is −160 dBW. For accurate measurements, a minimum SNR of 20 dB is needed. When we assume that fading minima of −35 dB may occur, the system has to be designed for a mean input power of −160 + 20 + 35 = −105 dBW. The antenna gain at both ends of the link is 3.7 dBi. The ionospheric losses are low: we estimate them at 4 dB. The path length through the ionosphere can be approximated by assuming an ionospheric refraction height of 220 km and a horizontal distance of 100 km. A wave travelling in a straight line to the reflection point would cover a path length *R* of:

(7) $$R=2\sqrt{220\;km^{2}+{\left(\frac{150\;km}{2}\right)}^{2}}=451\;km$$

As the real path is a curved and not a straight line, approximately 1.2% must be added to its length, so that we arrive at a path length of 457 km. Probe frequency is 7 MHz. Using the Friis transmission equation for free space propagation [39] complemented with the ionospheric losses, we can calculate the required probe transmit power:

(8) $$P_{probe}=P_{sensor}-G_{sensor}-G_{probe}+20\log_{10}R-20\log_{10}\left(\frac{c}{4\pi f}\right)+A_{i}$$

where *P_probe_* and *P_sensor_* are in dBW and *G_probe_* and *G_sensor_* are the antenna gains in dBi and *A_i_* are the ionospheric losses in dB. *c* is the speed of light (2.9979 × 10^8^ m/s) and *f* is the frequency in Hz. Substituting these values in (9), the minimum power that the probe must deliver to the antenna is:

(9) $$P_{probe}=-105-3.7-3.7+20\log_{10}\left(457\times 10^{3}\right)-20\log_{10}\left(\frac{2.9979\times 10^{8}}{4\pi *7\times 10^{6}}\right)+4=-5.9\;\mathrm{dBW}$$

According to these calculations, a minimum probe power of 0.26 Watt will produce 55 dB SNR at the sensor in a 30 Hz bandwidth. When the probe power is increased to 1 Watt, the sensor power will become −99 dBW, and the SNR in a quiet rural environment will be 61 dB.

## 6. Probe Design

**Figure 6:**
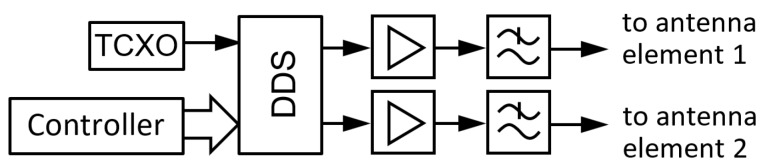
The block diagram of the probe signal transmitter.

For the investigation, a sequence of probing signals of precisely controlled polarization are needed. This can be realized by driving the two orthogonal antenna elements with two sine waves that have the same frequency, but a different amplitude and phase. The polarization of the emitted wave can be controlled by changing the power ratio *ρ* and the phase difference *θ* of the two outputs of the probe:

(10) $$k_{AR}=\sqrt{\frac{1+\rho +\sqrt{1+\rho^{2}+2\rho \;\cos\left(2\theta \right)}}{1+\rho -\sqrt{1+\rho^{2}+2\rho \;\cos\left(2\theta \right)}}}$$

where *k_AR_* is the axial ratio of the polarization ellipse [40]. To control the power ratio *ρ* and phase difference *θ* a novel dual-channel transmitter is designed around a direct digital synthesis (DDS) chip. The DDS generates two synchronous sinusoidal voltages, derived from a single 25 MHz temperature compensated crystal oscillator (TCXO) with a frequency stability of 2.5 × 10^−6^. The two outputs of the DDS are followed by two analogue 1-Watt amplifiers and low pass filters, as shown in Figure 6. The relative amplitude and phase difference of the signals sent to antenna 1 and antenna 2 can be controlled by writing a digital word in a register of the DDS chip. To achieve circular polarization with >25 dB cross-polarization, the power error must be smaller than 0.3 dB and the phase error must be smaller than 2.5° [25]. Therefore, any amplitude and phase errors introduced by the analogue stages are measured and a calibration factor is stored to compensate for them. Similarly, the frequency of the probes is measured and calibrated by storing a digital multiplication factor.

**Figure 7:**
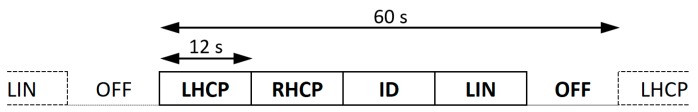
Example of a sequence of polarized probing signals. This example shows left-hand circular polarization (LHCP), right-hand circular polarization (RHCP), transmitter identification (ID) and linear polarization (LIN). The ‘off’ interval is used to synchronize probe and sensor.

A low-power programmable controller with non-volatile memory is integrated in the probe. With it, a sequence of different polarizations can be pre-programmed. An example of such a sequence, in which LHCP, RHCP and linear polarized (LIN) probing waves are alternated, is shown in Figure 7. Switching from one polarization to the other is done with a smooth slope to avoid wideband spurs or ‘clicks’ in the adjacent channels. The duration of the polarization intervals is derived from the TCXO frequency, making the interval length extremely precise and stable (2.5 × 10^−6^). As the frequency of the transmitted wave is also derived from the same TCXO, the frequency is precisely known at the receiver and with it the interval length. The sensor can now be synchronized with the polarization intervals of the probe by detecting the ‘off’ interval that is inserted in each sequence. The controller will also be used to activate the probes on a programmable day and time and deactivate them at night to save battery power. Remote control via the mobile phone network has been investigated but the resulting power consumption would be too high for this application. 

The entire probe transmitter is mounted in a 23 cm × 8 cm × 8 cm water-tight box at the masthead, in the centre of the antenna system. This eliminates the use of coaxial antenna cables and calibration of their length and also reduces weight. A single lightweight telescoping mast is used to support the masthead, from which the antenna wires slope downward in four directions. A mast-foot makes the mast self-supporting during installation. The antenna wires also act as guy wires. The battery at the base of the antenna mast provides counterweight and a 12 VDC cable runs up the mast to feed the transmitter. The complete probe can be installed by one person in 2 h, which is important for the deployment of all the probes in a short period of time in a wide area. Eight probes are realized in total. Together with an equally portable sensor system, an accurate and deployable system is realized to investigate the influence of the ionosphere and the Earth’s magnetic field on radio wave propagation below the plasma frequency of the ionosphere.

## 7. Deployment Criteria

As the polarization purity of the waves emitted by the probes and the cross-polarization of the sensor are essential in the intended experiments, the probes and sensors have to be deployed in environments that do not distort the emitted or received waves. This means that the near-field of the antenna, up to 1 or 2 wavelengths (40–80 m at 7 MHz), should be devoid of metal wiring (wire fences, power cables, telephone wire) and large conducting objects. Furthermore, the ground under the antenna wires, which is also in the near-field and influences the antenna diagram, must be homogeneous and flat. Rooftops are generally not suitable, as the structure directly under the antenna may contain conducting supports and wiring, which will distort the antenna diagram. In the far field, resonant objects (e.g., HF antennas) or multi-resonant objects (e.g., telephone and power lines) should preferably not be within line-of-sight. In practice, conforming to all these criteria is not always possible, but the more strictly these criteria are adhered to, the higher the quality of the measurements.

**Figure 8:**
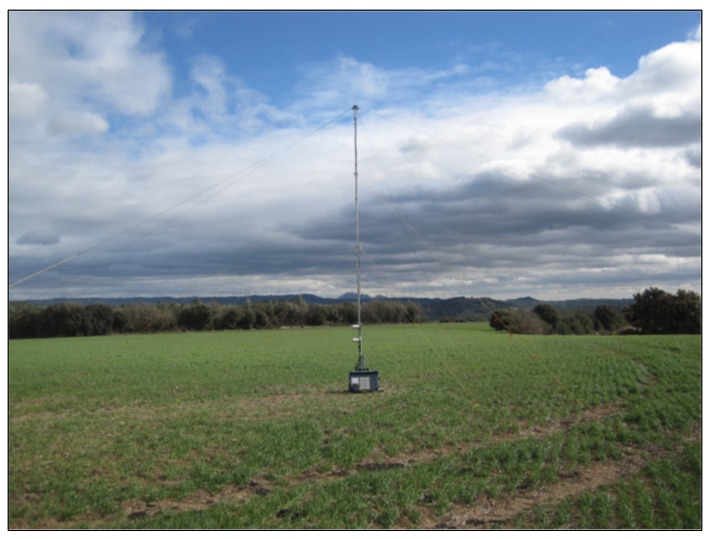
The central sensor system in in Sant Martí Sesgueioles.

**Figure 9:**
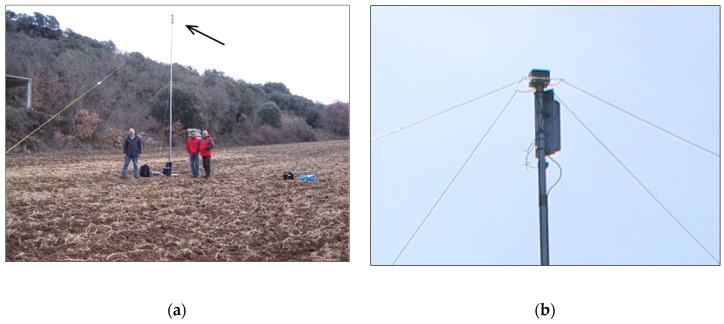
One of the six probe transmitters in Cal Cerdanyola. (**a**) The complete system including the wire antennas; (**b**) A close up of the compact (23 cm × 8 cm × 8 cm) probe transmitter on top of the mast.

Additionally, for the sensor, a location where the radio noise level is very low has to be selected. City locations generally exhibit very high radio noise levels. Photovoltaic sites may also emit high levels of radio noise. Radio noise is generally lowest in remote rural areas, with a very low density of buildings and human activity. As the waves that we observe in the NVIS experiments arrive at very steep angles, typically 70°–90°, a measurement site located in a valley and shielded from far-away noise sources by mountains may be preferred over hilltop sites with a good view all around. Radio noise measurements with calibrated equipment are preferred to assess the local radio noise level. An example will be given for the measurements in Spain. Firstly, the central low noise sensor location was selected and verified by measurement. Secondly, potential probe locations were identified at distances between 50 and 150 km from the sensor location via friends and colleagues. Screening of potential sites was done firstly via Google Earth, then secondly by personal inspection. Figure 8 and Figure 9 provide an impression of such sites. The site in Cal Cerdanyola was located in a canyon in the lower Pyrenees, with steep mountain ridges all around. This site provided an excellent demonstrator of the performance of NVIS radio systems in areas seemingly cut off from the horizon.

## 8. Validation Measurements

Measurement data obtained during a field deployment of six probes and one sensor in Spain was analysed to verify the performance of the design described here. This is not the science performed with the system—which requires a different type of analysis and will be subject to separate publications—but a validation of the realized system of sensor and probes. 

**Figure 10:**
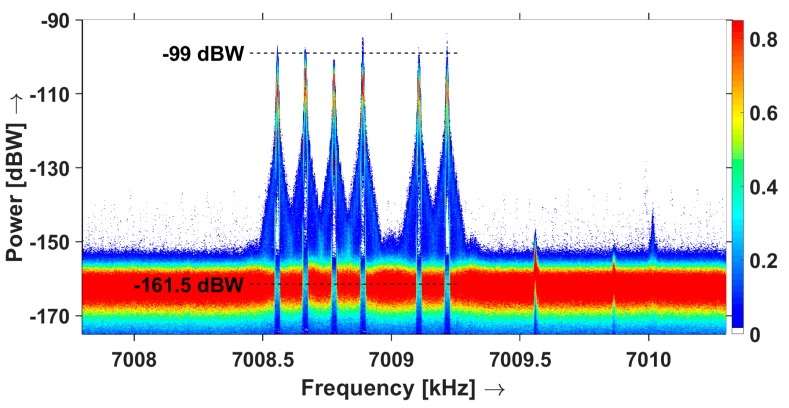
Statistical spectrogram showing the six probe signals as received by the sensor. The colour shows the relative occurrence in a 0.15 dB amplitude bin and a 3 Hz frequency bin.

In our design, we assumed a radio noise level of -160 dBW in a 30 Hz bandwidth, and expected a signal level of -99 dBW and a resulting SNR of 61 dB. We processed 45 min of data from the field deployment using a Fast Fourier Transformation (FFT) [41] with a block size of 1000 samples and a Blackman-Harris window [42] and a bin size of 3 Hz. We than aggregated the processed data in a statistical spectrum plot, in which the colour represents the relative occurrence of each of the values, see Figure 10. The 6 peaks correspond with the six probe signals. Other amateur radio signals can be seen around 7009.57, 7009.86 and 7010.05 kHz. As they do not interfere with our measurements, no effort is undertaken to identify them. From this graph, we can see that the maximum probe signal power is approximately −99 dBW, with a few dB difference per probe. However, the radio noise level is approximately −161.5 dBW in a 3 Hz bandwidth, or −151.5 dBW in a 30 Hz bandwidth. This is 8.5 dB higher than was predicted for a quiet rural environment. As a result, the SNR is only −99 – (−151.5) = 52.5 dB. The resulting SNR is sufficient for the intended experiments, but the bandwidth may be decreased to 10 Hz, which will improve the SNR to 57.3 dB.

**Figure 11:**
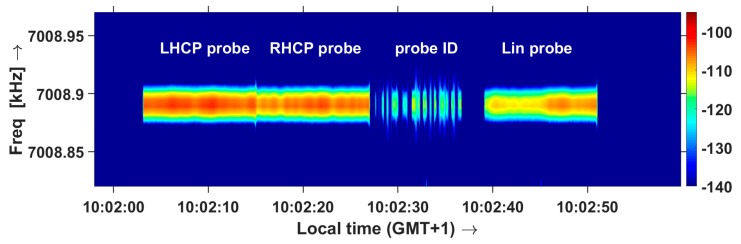
Spectrogram of the LHCP end RHCP waves emitted by the probe and received by the sensor on a linearly polarized antenna, during normal NVIS propagation.

**Figure 12:**
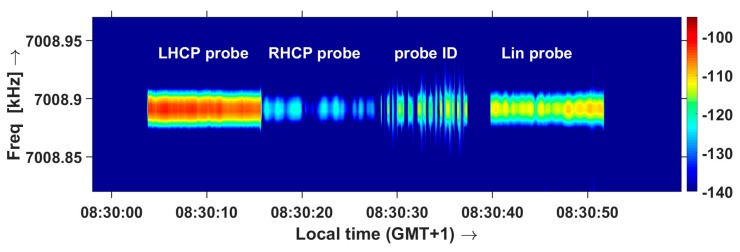
Spectrogram of the LHCP end RHCP waves emitted by the probe and received by the sensor on a linearly polarized antenna, during the morning Happy Hour.

Cross-polarization of the probes is verified during ‘Happy Hour’ propagation [25], during which the ionosphere acts as a polarization filter and only the extraordinary wave propagates. Figure 11 shows the spectrogram of the waves received by the sensor on a linearly polarized antenna during normal NVIS propagation, showing that the waves emitted in LHCP and RHCP are received equally strong. Figure 12 shows reception during the Happy Hour interval, and 25–35 dB suppression of the ordinary wave (RHCP transmission of the probe). This proves that the cross-polarization of the probe is very good. During the field tests, one of the probes developed a faulty amplifier in one channel. This was immediately visible at the sensor, as the transmit cross-polarization was lost. 

**Figure 13:**
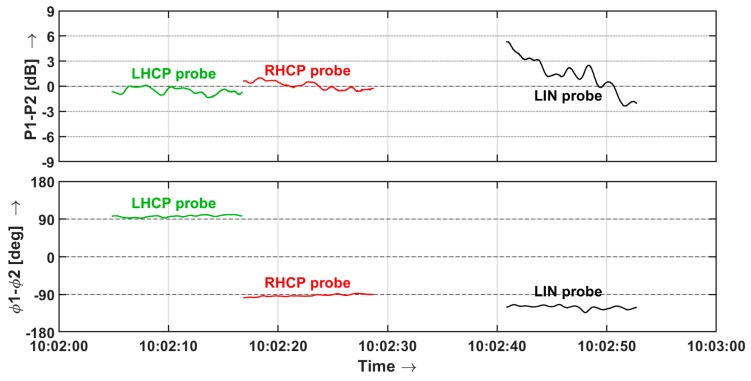
Polarization of a sequence of probing signals captured by the sensor during normal NVIS propagation.

The performance of the probe can to some extent be extrapolated to the sensor, as identical antennas are used and the receiver has been characterized using laboratory equipment. Furthermore, the field measurement makes use of hourly calibrations with a precise dual channel source and shows very little drift. The results obtained with stable NVIS propagation are promising, see Figure 13. The first two intervals show almost identical power in both polarization planes, but +90° and −90° phase difference respectively, corresponding with received RHCP and LHCP polarization. This is consistent with the extraordinary and ordinary wave propagation and the reversal of the polarization when the waves are refracted downward [6,32]. The interval in which the probe sends linearly polarized waves clearly shows rapidly changing polarization caused by interference of the ordinary and extraordinary wave, which also concurs with expectations.

## 9. Discussion and Conclusion

The validation measurement and the field trials have shown that an exceptionally portable and very sensitive and accurate measurement system is created. The 1-Watt probe signal transmitters produce a 52.5 dB SNR in a 30 Hz bandwidth. This is 8.5 dB lower than predicted, caused by a higher than expected level of external radio noise. The radio noise level shows a diurnal variation consistent with NVIS ionospheric propagation, which could indicate that electromagnetic noise from the city of Barcelona, at 100 km distance, is received via ionospheric refraction. This would be consistent with the work of Pederick and Cervera [43] on radio noise propagation via the ionosphere and with our observations of significant radio noise reduction during a Sudden Ionospheric Disturbance [28], but cannot be proven without directional information.

The polarization purity of the waves emitted by the probes is excellent. To the best of our knowledge, there are no other published concepts that reach comparably high cross-polarization values (25–35 dB) in HF transmission, which is much better than the cross-polarization achieved by Erhel et al. with large fixed antenna systems [24]. The fully digitally generated polarization, calibration and automated polarization sequence transmission provide unequalled experimental flexibility. 

The validation measurements of the sensor conform with expectations and show realistic propagation characteristics and variations. To objectively establish the cross-polarization of the sensor, a drone-based source would be required. The active antennas of the radio astronomy ‘LOw Frequency ARray’ (LOFAR) [44] provide >24 dB cross-polarization rejection for linearly polarized waves [44]. However, the measurements described by Paonessa, et al., which are the current state-of-the-art, can only provide cross-polarization for linearly polarized antennas and with some orientation uncertainty [45]. Drone measurement systems for circular polarization are not available yet, but are certainly on our wish-list. 

Compared with our previous measurement system, which was built around a professional measurement receiver, the measurement speed is >6000 times higher, the sensitivity is >10 dB better and the IFDR is >20 dB better. The sensor no longer needs switched input filters to avoid intermodulation. For comparison, the LOFAR active antennas produce intermodulation products from the strongest HF signals and high-pass filters are introduced to limit reception to frequencies above 20 MHz. Where the previous system could only alternatingly measure LHCP and RHCP, the novel system can reconstruct an infinite number of polarizations in parallel in each sampling moment.

Quantitative comparisons with other systems were not undertaken; currently there are no systems that could do the same measurements. LOFAR could be used for the sensor side, but the presence of high-pass filters makes the reception of the low-power probes improbable.

This work has led to the creation of a novel instrument and several new concepts are introduced that will contribute to future ionospheric propagation research. Interesting future extensions to the system could be time-of-flight measurements to investigate multipath and directional information to investigate origins of radio noise and of night-time scatter.

## Acronyms

DDS	Direct Digital Synthesis
EurAAP	European Association on Antennas and Propagation
FFT	Fast Fourier Transform
HF	High Frequency (3-30 MHz)
HPSDR	High Performance Software Defined Radio
IFDR	Intermodulation Free Dynamic Range
IRI	International Reference Ionosphere
ITU	International Telecommunication Union
IGRF	International Geomagnetic Reference Field
LHCP	Left-Hand Circular Polarization
NEC	Numerical Electromagnetics Code
NVIS	Near Vertical Incidence Skywave
RHCP	Right-Hand Circular Polarization
SNR	Signal-to-Noise Ratio
TCXO	Temperature Compensated Crystal Oscillator
